# NMJ Analyser: a novel method to quantify neuromuscular junction morphology in zebrafish

**DOI:** 10.1093/bioinformatics/btad720

**Published:** 2023-12-07

**Authors:** Jaskaran Singh, Yingzhou Edward Pan, Shunmoogum A Patten

**Affiliations:** Institut National de la Recherche Scientifique (INRS), Centre Armand Frappier Santé Biotechnologie, Laval, QC H7V 1B7, Canada; Institut National de la Recherche Scientifique (INRS), Centre Armand Frappier Santé Biotechnologie, Laval, QC H7V 1B7, Canada; Institut National de la Recherche Scientifique (INRS), Centre Armand Frappier Santé Biotechnologie, Laval, QC H7V 1B7, Canada; Département de Neurosciences, Université de Montréal, Montreal, QC, Canada

## Abstract

**Motivation:**

Neuromuscular junction (NMJ) structural integrity is crucial for transducing motor neuron signals that initiate skeletal muscle contraction. Zebrafish has emerged as a simple and efficient model to study NMJ structural morphology and function in the context of developmental neurobiology and neuromuscular diseases. However, methods to quantify NMJ morphology from voluminous data of NMJ confocal images accurately, rapidly, and reproducibly are lacking.

**Results:**

We developed an ImageJ macro called “NMJ Analyser” to automatically and unbiasedly analyse NMJ morphology in zebrafish. From the Z-stack of a zebrafish hemisomite, both presynaptic and postsynaptic fluorescently labeled termini at NMJs are extracted from background signal, with larger clusters of termini being segmented into individual termini using an unbiased algorithm. The program then determines whether each presynaptic terminus is co-localized with a postsynaptic terminus and *vice versa*, or whether it is orphaned, and tabulates the number of orphan and co-localized pre- and postsynaptic termini. The usefulness of this ImageJ macro plugin will be helpful to quantify NMJ parameters in zebrafish, particularly during development and in disease models of neuromuscular diseases. It can enable high-throughput NMJ phenotypic screens in the drug discovery process for neuromuscular diseases. It could also be further applied to the investigation of NMJ of other developmental systems.

**Availability and implementation:**

NMJ Analyser is available for download at https://github.com/PattenLab/NMJ-Analyser.git.

## 1 Introduction

Functional and anatomical studies of the neuromuscular system among the vertebrates have formed the basis of our understanding of motor neuronal control of muscle contraction and locomotion (D’Elia and Dasen 2018, Luxey *et al.* 2020, Xu *et al.* 2022). The zebrafish model has proven to be a powerful tool for studying the vertebrate neuromuscular system. It is a widely used model organism in scientific research due to its advantageous features, such as high transparency and external development of embryos. These features make zebrafish an ideal candidate for microscopic imaging techniques, such as confocal, two-photon, and super-resolution microscopy. These non-invasive imaging techniques in the zebrafish provide researchers with the ability to visualize the internal anatomy, development, and behavior of the organism at the cellular and molecular levels. Furthermore, the genetic similarity between the zebrafish and humans and the ease of genetic manipulation of the zebrafish genome have made it a powerful tool for understanding human biology and disease (Howe *et al.* 2013). In addition, the ability to perform large-scale genetic screens in the zebrafish makes it an effective tool for identifying novel disease genes and studying their function. The ability to perform high-resolution imaging in a living organism has revolutionized the field of biological research, and the zebrafish is playing a critical role in advancing our understanding of the underlying mechanisms of development and disease. Several studies have capitalized on the advantages of the zebrafish and provided important insights in motor axonal growth (Myers *et al.* 1986, Chen *et al.* 2012, Sainath and Granato 2013), neuromuscular junction (NMJ) development (Jing *et al.* 2010, Bailey *et al.* 2019), synaptogenesis (Jontes *et al.* 2000, Hutson and Chien 2002, Panzer *et al.* 2005, Son *et al.* 2020), muscle development (Skobo *et al.* 2014, Dubińska-Magiera 2016, Chen *et al.* 2017, Yin *et al.* 2022), and in NMJ deficits underlying neuromuscular pathologies (Patten *et al.* 2017, Butti *et al.* 2021, Lescouzères *et al.* 2023). In the last few decades, the zebrafish has particularly emerged as a valuable tool in studying the pathogenesis and development of therapeutic interventions for various neuromuscular disorders (Singh and Patten 2022). Disturbances in NMJ morphology is one of the characteristic hallmarks of most neuromuscular diseases (NMDs) (D’Ydewalle *et al.* 2011, Martínez-Hernández *et al.* 2013, Butti *et al.* 2021). Various fluorescent reporter and transgenic zebrafish lines have been developed to model NMDs, which are used for high-throughput drug screening assays (Giacomotto *et al.* 2015, Patten *et al.* 2017, Singh and Patten 2022, Lescouzères *et al.* 2023). High-throughput studies involve evaluating the impact of thousands of potential drugs or toxic substances on NMJ morphology. Automated microscopic processes lead to the production of thousands of images and large amounts of data. This has led to a growing need for automated image processing and analysis to generate accurate results and eliminate the time consumption and biases in the manual analysis process. Unfortunately, the necessary image analysis goes beyond the capabilities of standard commercially available solutions, such as the software provided with microscopes or is confined to the specific format of images generated by some microscopes, which are available at higher costs and are not accessible to everyone. Thus, the creation of a specialized image processing technique for zebrafish NMJ analysis is critical in realizing the full potential of the data contained in the images collected.

**Figure 1.**
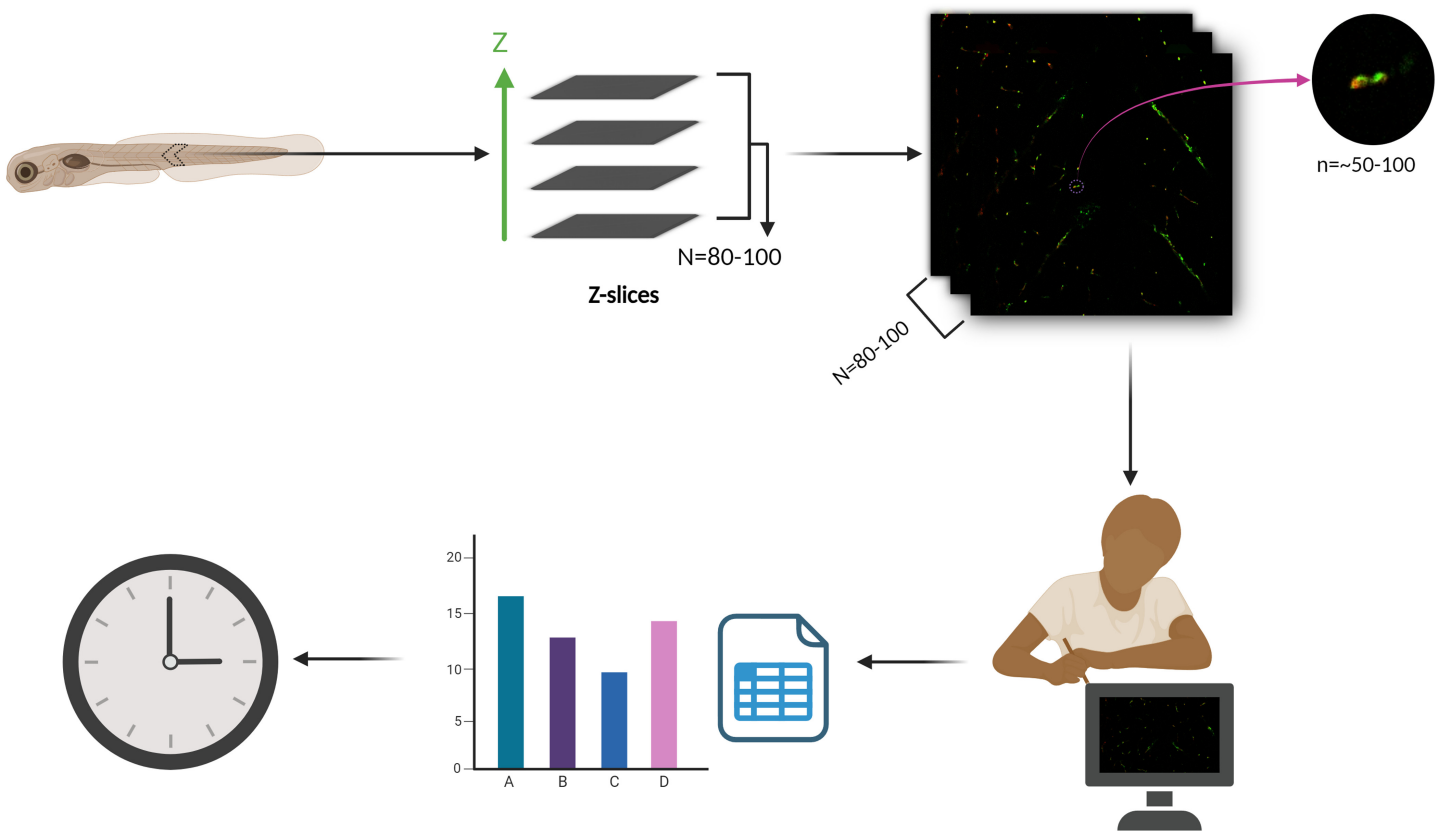
Manual analysis of NMJ morphology is time-consuming and prone to bias. Confocal imaging of zebrafish NMJs, stained with presynaptic marker SV2a and postsynaptic marker α-bungarotoxin (α-BTX), entails acquisition of 80–100 z-slices (*N*). Each z-slice contains an estimated 50–100 synaptic puncta (*n*). The manual quantification of individual puncta within separate channels (2) and the assessment of their co-localization necessitate a substantial investment of time and effort, particularly when analyzing multiple samples representing diverse experimental conditions and varying time points. Moreover, limitations in the resolution of confocal microscopy introduce the possibility of under or overestimation of synaptic puncta counts.

Zebrafish NMJ images acquired via confocal microscopy are typically large, which makes processing, and analysis a hefty task (Fig. 1). For instance, analyzing the NMJ morphology in a single 6 days postfertilization (dpf) zebrafish requires at least two channels to label the pre- and postsynapse. To obtain a comprehensive z-stack, at least 80–100 slices per channel must be taken at 40× magnification, with an optimal slice distance of ∼0.44 µm. This results in nearly 300 slices (presynapse + postsynapse + merged) with numerous puncta on each slice to be analyzed. Furthermore, when multiple zebrafish are analyzed for various drug treatments at various time points, these numbers rapidly increase, making the analysis process extremely complex and difficult.

To address this hefty task, we developed an automated image analysis program specifically focusing on the analysis of zebrafish NMJ morphology. In order to make our method more accessible and applicable, we have integrated it into an open-source image analysis software Fiji (Schindelin *et al.* 2012). Fiji provides a custom graphical user interface and comprehensive documentation to guide users through the process. An automated workflow pipeline has been established for accessing confocal zebrafish NMJ files to create z-projections. Users are able to define their region of interest (ROI) within the z-projection, typically a hemisomite region, and an automated analysis will be run on multiple channels for each defined hemisomite in the image. The analysis results for each hemisomite include: (i) the number of presynaptic puncta, (ii) the number of postsynaptic puncta, (iii) the number of presynaptic puncta co-localizing with postsynaptic puncta, and (iv) the number of postsynaptic puncta co-localizing with presynaptic puncta. Images of zebrafish NMJ obtained through confocal microscopy frequently exhibit clustering of puncta due to limitations in resolution, resulting in inconsistent manual counting of puncta and biased co-localization analysis. This can compromise the accurate analysis of NMJ morphology, which is crucial in the study of many NMDs. To address this challenge, we have developed an automated image processing approach that incorporates an unbiased segmentation algorithm to detect and segment clustered puncta. Our segmentation algorithm is based on the analysis of three parameters: area, circularity, and aspect ratio, to accurately identify clustered puncta. Our program will enable users to perform high-throughput, high-accuracy data analysis in a short time frame through an unbiased approach.

## 2 Materials and methods

### 2.1 Zebrafish maintenance

Wild-type (AB/TL strain) (*Danio rerio*) and C9orf72-KD (C9-miR) amyotrophic lateral sclerosis (ALS) model (Butti *et al.* 2021) were maintained at 28°C at a light/dark cycle of 12/12 h in accordance with Westerfield zebrafish book (Westerfield 2000). Embryos were raised at 28.5°C, and collected and staged using standard criteria. All experiments were performed in compliance with the guidelines of the Canadian Council for Animal Care and the local ethics committee of INRS.

### 2.2 Immunohistochemistry

Zebrafish 6 days postfertilization (dpf) larvae were fixed in 4% paraformaldehyde in phosphate buffered saline (PBS) at 4°C overnight. The next day, the fish were rinsed 3× in a 0.1% Tween-20 in PBS solution (PBS-Tween) for 15 min at room temperature. The fish were then permeabilized in 1 ml of 1 mg/ml collagenase (Sigma-Aldrich; C0130-100MG) in PBS solution for 2.5 h at room temperature on a rotator. The fish were then rinsed three times with PBS-Tween for 15 min at room temperature on a rotator. The larvae were placed for 1 h at room temperature in blocking solution (1% bovine serum albumin, 1% DMSO, 1% Triton-X, 2% normal goat serum, in PBS). They were then incubated in a (10 mg/ml) tetramethylrhodamine-conjugated α-bungarotoxin (Thermofisher T1175) in 0.1% PBS-Tween for 30 min to stain the postsynaptic acetylcholine receptors. The larvae were rinsed several times with 1× phosphate buffer with 0.1% Tween 20 for 30 min and then incubated in freshly prepared blocking solution containing primary antibody SV2 (1:200, Developmental Studies Hybridoma Bank) overnight at 4°C. The next day, the fish were rinsed three times with PBS-Tween for 15 min at room temperature on a rotator before being incubated in a 1:1000 Alexa Fluor 488 goat anti-mouse (Invitrogen; A10680) in blocking buffer for 4 h at room temperature on a rotator. The fish were then rinsed three times with PBS-Tween for 15 min at room temperature on a rotator before being stored in PBS-Tween overnight at 4°C without rotation. The larvae were then mounted the next day in fluoromount-G (Invitrogen 00–4958-02) on a slide and imaged using Zeiss LSM 780 confocal microscope (Carl Zeiss, Germany). The fish were imaged in Z-stacks with a xy-resolution of 1024 × 1024 at 0.21 µm per pixel and a z-resolution of 0.44 µm.

### 2.3 Software

The macro presented in this program is written in the ImageJ macro language using the macro editor in FIJI.

## 3 Results

### 3.1 Workflow for NMJ morphology analysis and quantification

**Figure 2.**
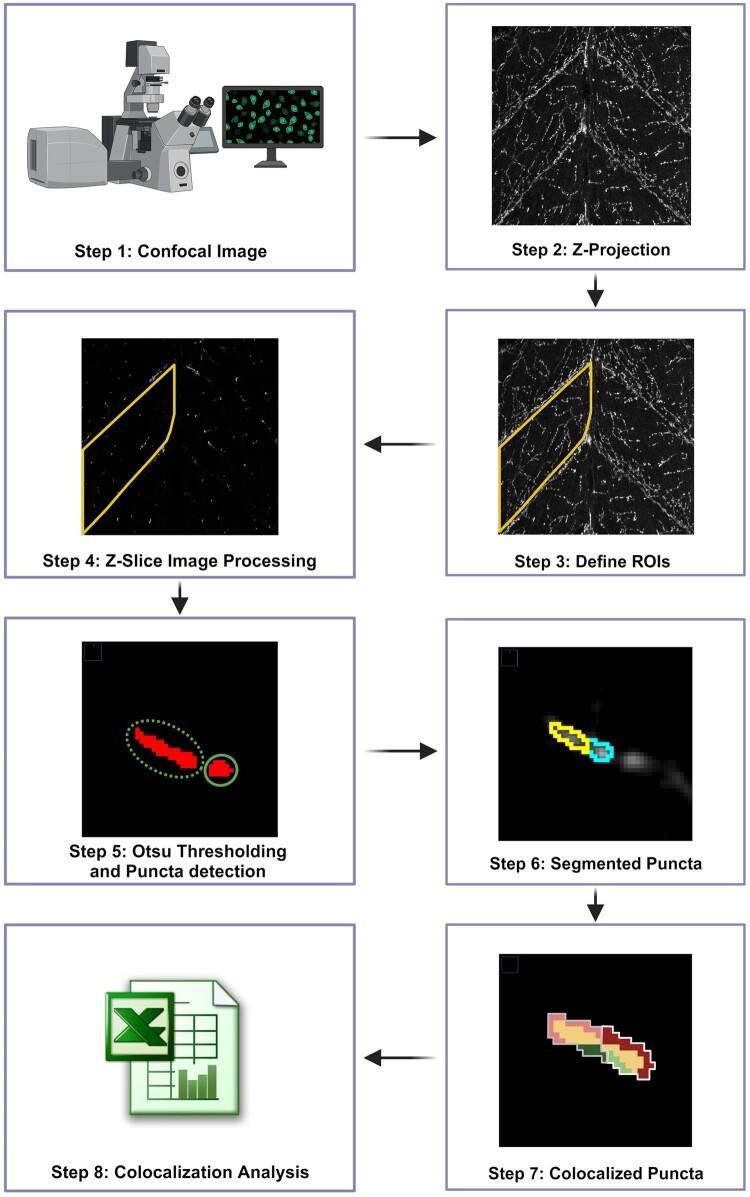
NMJ analysis workflow overview (Steps 1–7). Z-projection are created of the confocal images where users are prompted to define the ROIs. Each z-slice is processed for contrast enhancement and punctum detection via Otsu thresholding. Clustered puncta are segmented into single punctum and co-localization analysis is performed for each punctum in the defined ROI.

The automated image analysis program is designed to analyse confocal images and identify synaptic puncta within the images (Fig. 2). The program begins by opening the confocal images from a user-selected folder and generating a z-projection for each file present in that folder (Supplementary Video S1). The user is then prompted to define the ROI, which is saved in a specified folder. The program then saves the number of slices present in each image for each channel and performs this process for all the files present in the selected folder. Next, each slice for each channel is opened and processed with a series of image processing parameters.

The first step in image processing is to apply a Gaussian blur with a sigma of 1.0 to the image. To enhance contrast and detect puncta, the histogram of the image is expanded. This is achieved by analyzing the minimum and maximum grayscale values of the image slice, then increasing the minimum and decreasing the maximum grayscale values by 10% of the dynamic range (max. to min. grayscale value) of the image. After histogram expansion, Otsu thresholding (Otsu 1979) is applied to the image slice. The image generated by Otsu thresholding is then despeckled by applying an area parameter of 0.043 µm^2^ and removing all pixels selected by Otsu that have an area of 0.043 µm^2^, to filter out noise.

The program uses a Fiji plugin to analyse particles and store the saved ROIs of each particle (which represents a puncta) detected in the slice. By manual analysis of multiple single and clustered puncta, we identified three parameters to which can differentiate clustered puncta from individual puncta; area, circularity, and aspect ratio. Any particle detected by Otsu whose area is >4 µm^2^, circularity is <0.65, and aspect ratio is >2.5 is considered to be a clustered punctum and is further selected for segmentation.

Our automated analysis program reopens the image slice and the ROIs of the clustered puncta for that particular image slice. A Gaussian blur with a sigma value of 1.0 pixel is applied to the image slices. The ROIs marked as clustered puncta are selected from the slice and the “Find Maxima” command is run. If more than one “maxima” are reported within a clustered punctum, the *x* and *y* coordinates of the “maxima” are recorded. Then, a new binary image of the same dimension is created. The maximum coordinates in the new binary image are assigned a grayscale value unique to each “maxima”. The program then executes an unbiased expansion algorithm to segment the puncta into *n* parts, where *n* is the number of “maxima” reported. The expansion continues until the initial ROI is completely distributed among the *n* maxima. The segmented puncta are then filtered based on their area, circularity, and aspect ratio. If the area is smaller than 4 µm^2^, the circularity is >0.65, and the aspect ratio is smaller than 2.5, the segmented puncta are considered to be good/single puncta. If the conditions are not met, the puncta are further segmented until they meet the parameters. After the segmentation process, good puncta from each slice and each channel of each image file are used for co-localization analysis. The program creates two binary images of the same image slice number for Channels 1 and 2, with the puncta of each slice for their respective channel. The Boolean function AND is run on both slices belonging to Channels 1 and 2, and the number of single, co-localized, and total puncta is reported in a tabular form for each hemisomite or ROI for each image. A punctum is considered to be individual if the percent of its area covered by a punctum of the other channel falls below a specific threshold and to be co-localized if the covered area percent falls above a specific threshold. The result table reports the population of individual and co-localized punctum with progressively higher thresholds, from >0% to =100% in increments of 10%, resulting in 11 thresholds.

### 3.2 Image processing and thresholding

After the ROI has been defined in the respective images, the slices of the z-stack for each channel and each image are stored in their corresponding folders. Subsequently, each slice for each channel is subjected to image processing parameters. The first step in image processing involves applying a Gaussian blur with a sigma of 1.0 to the slice. Gaussian blur is often used in scientific image processing to reduce image noise and smooth out image intensity variations, which can help improve the results of subsequent image analysis steps. The Gaussian blur works by convolving the image with a Gaussian filter, which has a bell-shaped intensity distribution. This convolution effectively replaces the intensity values of each pixel with a weighted average of its neighbors, which has the effect of smoothing out sharp intensity transitions and reducing noise. By using a Gaussian blur, the features of interest in the image slice, such as puncta, can become more apparent and easier to detect.

**Figure 3.**
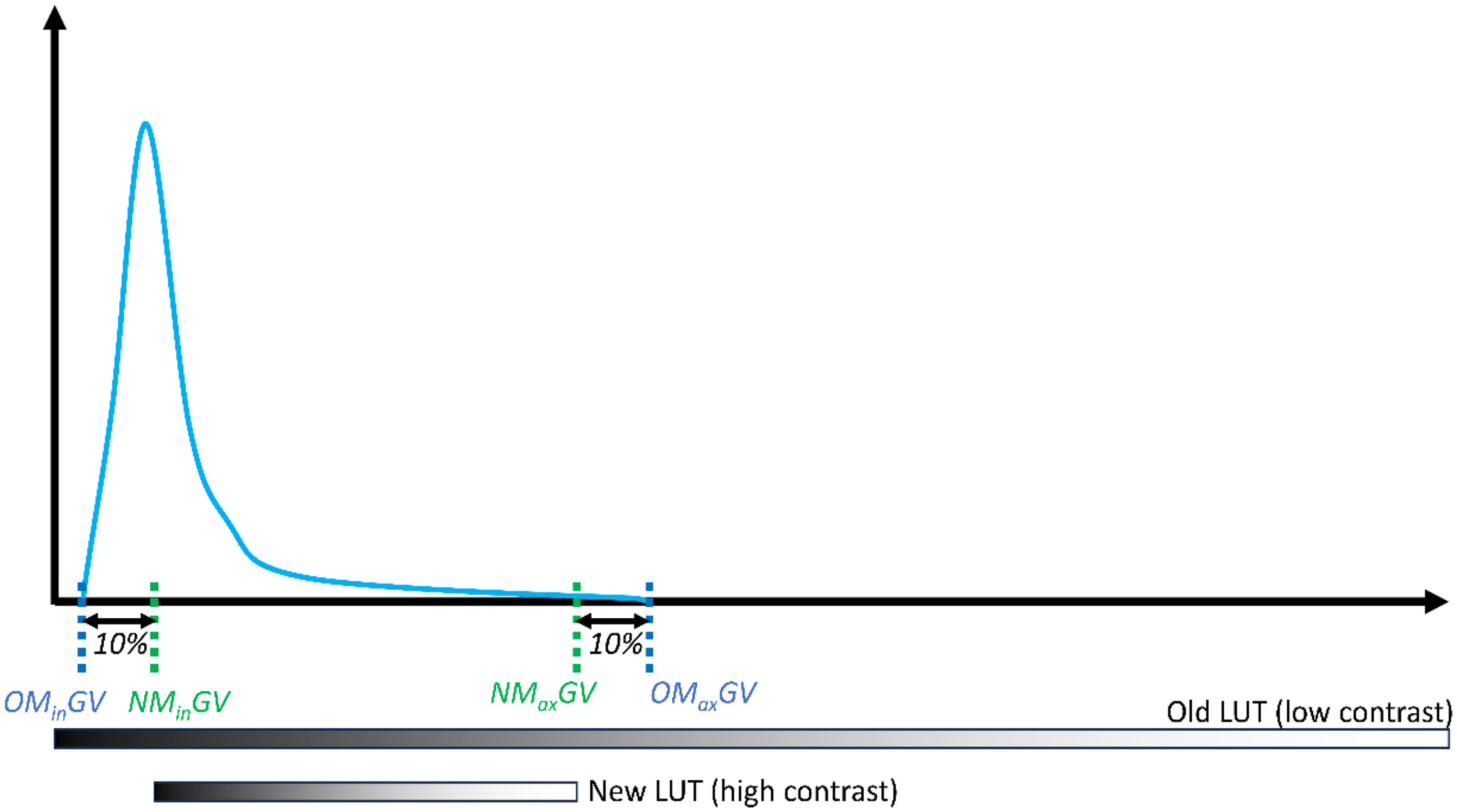
Distribution curve demonstrating histogram LUT compression. Increasing the minimum and decreasing the maximum grayscale values by 10% of the dynamic range (max. to min. grayscale value) of the image slice increases the contrast of the image for puncta detection. NM_in_GV: New Minimum Grayscale Value, NM_ax_GV: New Maximum Grayscale Value, OM_in_GV: Original Mininum Grayscale Value; OM_ax_GV: Original Maximum Grayscale Value.

Then, we utilized histogram expansion to enhance the image's contrast while avoiding the saturation of pixels. This approach allows for the detection of puncta through Otsu thresholding in Fiji. In the formula we developed for histogram expansion, the minimum and maximum grayscale values of the image are analyzed, and the extreme 10% of the grayscale values from the minimum and maximum pixel are trimmed out (Fig. 3). The histogram expansion (LUT compression) is calculated using the formula:


$$NM_{\mathrm{in}}GV=OM_{\mathrm{in}}GV+\frac{\mathrm{Range}}{10}$$

$$NM_{\mathrm{ax}}GV=OM_{\mathrm{ax}}GV-\frac{\mathrm{Range}}{10}$$

$$\mathrm{Range}=\;OM_{\mathrm{ax}}GV-\;OM_{\mathrm{in}}GV$$

$$NM_{\mathrm{in}}GV:\;\mathrm{New}\;\mathrm{Minimum}\;\mathrm{Grayscale}\;\mathrm{Value}$$

$$NM_{\mathrm{ax}}GV:\;\mathrm{New}\;\mathrm{maximum}\;\mathrm{Grayscale}\;\mathrm{Value}$$

$$OM_{\mathrm{in}}GV:\;\mathrm{Original}\;\mathrm{Minimum}\;\mathrm{Grayscale}\;\mathrm{Value}$$

$$OM_{\mathrm{ax}}GV:\;\mathrm{Original}\;\mathrm{Maximum}\;\mathrm{Grayscale}\;\mathrm{Value}$$

After processing the image with Gaussian blur and histogram stretching, Otsu thresholding (Otsu 1979) is applied to detect the puncta in the slice. Otsu is a computationally efficient and simple method to separate objects in an image based on intensity values. The method assumes that the image to be threshold contains two classes of pixels: foreground and background. The algorithm tries to find a threshold value and calculates the variance between the two classes and selects the threshold that maximizes the variance between the foreground and background pixels, which is equivalent to maximizing the separability between the two classes. The threshold is calculated based on the intensity histogram of the image, and the result is a binary image where the pixels with intensities above the threshold are set to one (foreground) and the pixels with intensities below the threshold are set to zero (background). This method is often used in scientific image processing to separate objects of interest from the background, making them easier to analyse.

### 3.3 Puncta analysis

**Figure 4.**
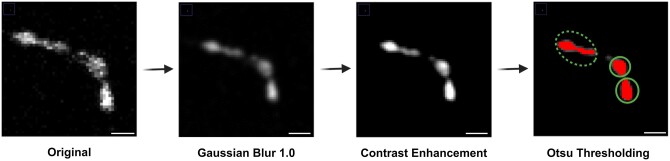
Image processing and Otsu thresholding for puncta detection. The original image was subjected to a Gaussian blur and contrast enhancement algorithm to enable the detection of puncta via Otsu thresholding. However, the limitations in resolution posed challenges in properly separating clustered puncta. The lack of resolution limits the clustered puncta to be separated. Dashed circle represents the clustered puncta; solid circle represents the single punctum. Scale bar = 2.5 µm.

After the detection of puncta by Otsu thresholding, there remains a limitation in the resolution of confocal microscopy that can lead to the detection of multiple clustered puncta as one punctum (Fig. 4). This creates a bias of overestimation or underestimation of the number of puncta in the image which can have a significant impact on the assessment of NMJ morphology. To mitigate this issue, we conducted a manual analysis of both single and clustered puncta in the image and identified three parameters that define clustered puncta. The three parameters to detect clustered puncta are:

1. Area >4 µm^2^
2. Circularity <0.65
3. Aspect ratio >2.5

Our automated analysis program applies these parameters to each punctum in the defined ROI for each z-slice and channel in the image. If any of these conditions are satisfied, the puncta are considered clustered and are further segmented by our unbiased segmentation algorithm until the parameters of a single punctum are met.

### 3.4 Segmentation

**Figure 5.**
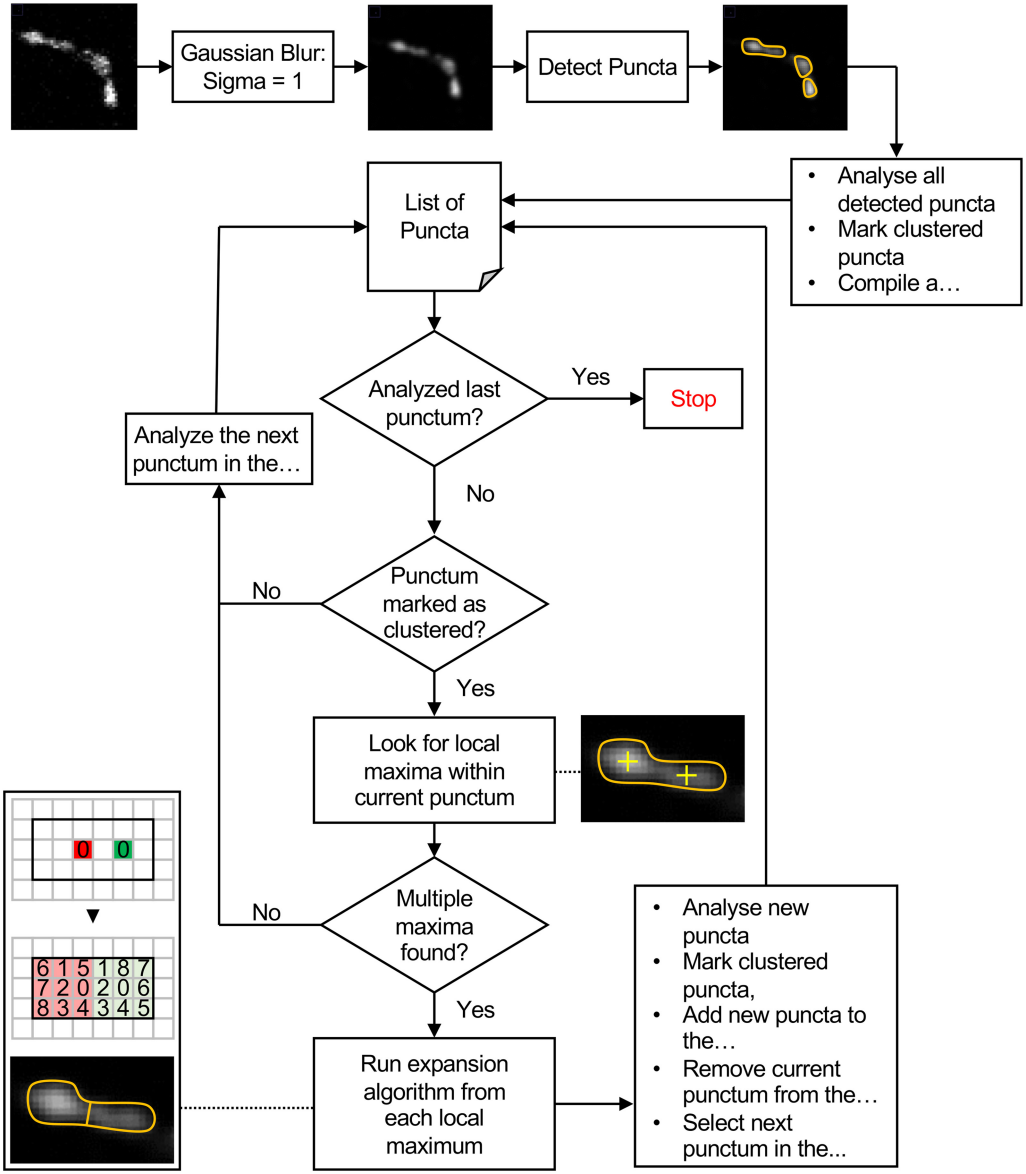
Flowchart of Segmentation Algorithm. The segmentation algorithm compiles a list of all detected puncta into a list, marking the clustered puncta. If a clustered punctum can be segmented, the segmented puncta are added to the list, the original punctum is removed from the list, and the next punctum in the list is analysed. If a punctum cannot be segmented or was not marked as a clustered, the next punctum in the list is analysed. These steps are repeated until all puncta have been analysed.

Any puncta marked as a clustered punctum will be further segmented into smaller puncta (Supplementary Video S2). The newly generated sub-puncta will recursively be analysed according to the Puncta analysis parameters described previously and segmented until they either fulfill all the criteria or the segmentation algorithm is unable to segment them further (Fig. 5).

The segmentation algorithm begins by applying a Gaussian blur with sigma = 1.0 pixels to the image. The program then goes down the list of puncta detected by puncta analysis and segments those marked as clustered puncta. The “Find Maxima …” ImageJ command is used to detect the presence of local brightness maximum within the punctum to be segmented. If no or a single maximum is found, then the cluster punctum is left as it is and the next punctum is analysed. If multiple “maxima” are detected within the cluster punctum, then the coordinates for each “maxima” is recorded in a list to be used as expansion nuclei in the expansion algorithm.

**Figure 6.**
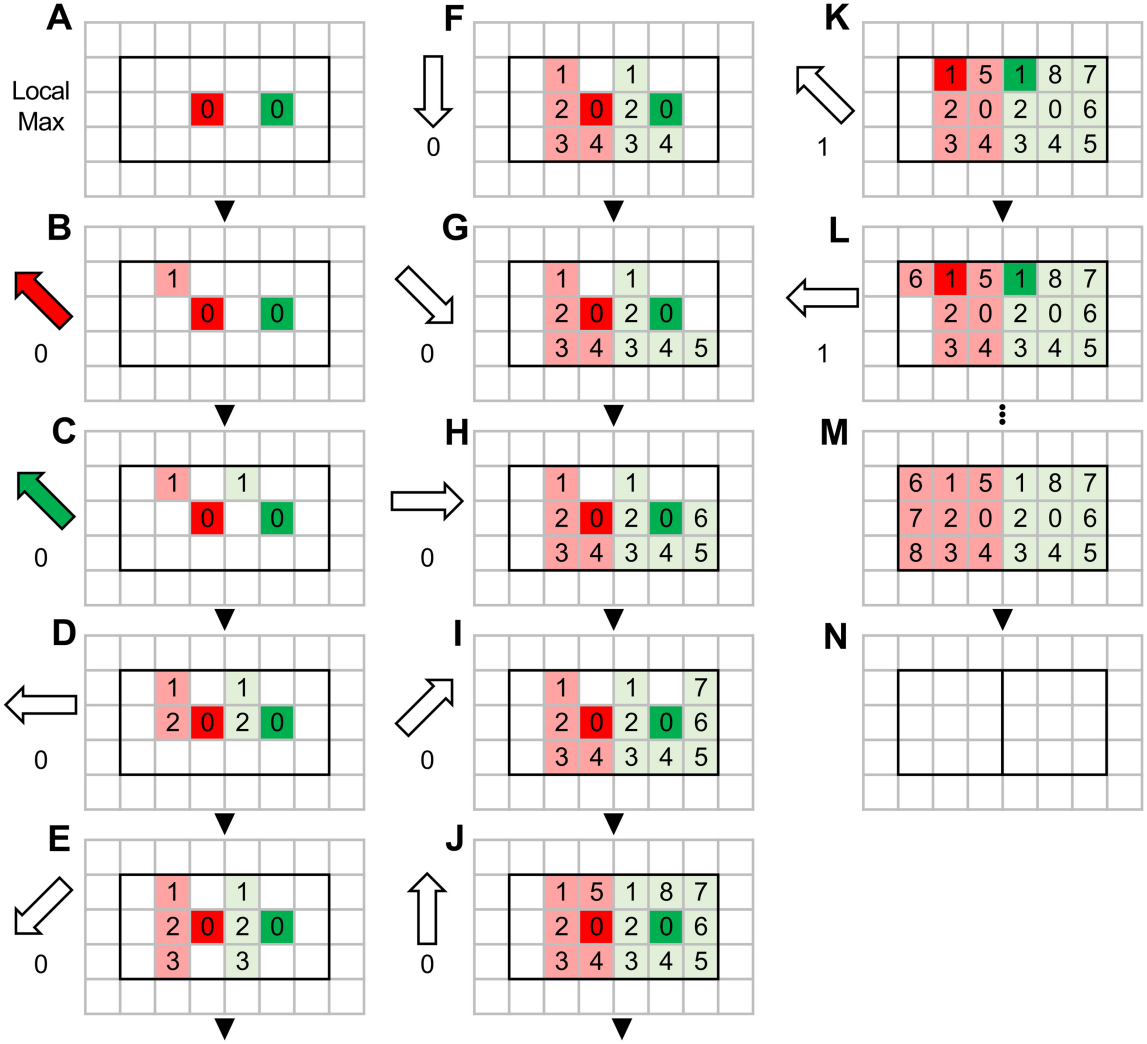
Expansion Algorithm. The expansion algorithm cycles through the eight neighboring pixels of each local maximum (A), beginning with top left (B and C), then cycling through each direction (D–J), assigning the neighboring pixel to a local maximum if the pixel is both unclaimed and within the punctum region. Once a cycle of eight directions has been completed, it is repeated with the first expansion as the origin of expansion (K). This process is repeated until the entire region of the punctum has been assigned to a local maximum (L–N).

The expansion algorithm uses the coordinates of each “maxima” as the point from which to expand each sub-punctum within the clustered punctum (Fig. 6A, Pixel 0 in red and green). It first checks whether the pixel to the top left of the first maxima is both empty and within the clustered punctum. If yes, the top left pixel is marked as part of the sub-punctum belonging to the first maxima and this pixel is added to the list of pixels associated with the first maxima (Fig. 6B, Red pixel 1). If the pixel is already taken by another maxima (Fig. 6G–I in red), then the pixel is not added to the list. The same process is performed for the other maxima in the clustered punctum (Fig. 6C, Green pixel 1). Subsequently, the pixel to the left, bottom left, below, bottom right, right, top right, and above pixel 0 for both maxima (red and green) are checked. Once this is complete, the algorithm will select the next pixel (Fig. 6K, Pixel 1, red and green) as the center from which to expand according to the same rules. The expansion algorithm stops when the area within the clustered punctum has entirely been distributed between the local maxima.

After the expansion algorithm stops, each contiguous region around each original local maxima is saved as a new punctum (Fig. 6M and N). The old clustered punctum is removed from the list of puncta while the new puncta are added to the list and flagged for a new round of segmentation analysis. The segmentation algorithm ends when it has checked every punctum on the list and either segmented them or determined that they cannot be segmented any further.

### 3.5 Co-localization analysis

As a final output, NMJ Analyser will tally up the total number of puncta detected in each channel and the number of puncta that do not overlap and the number of puncta that do overlap (co-localize) with puncta of the other channel. To determine whether a specific punctum is co-localized with the other channel, NMJ Analyser uses fractional overlap as the metric for co-localization. NMJ Analyser will measure the fraction of each punctum’s area that overlaps with the other channel and past a threshold of overlap, the punctum is considered to be co-localized. NMJ Analyser reports the fraction of puncta in both channels that are co-localized based on 11 thresholds: >0%, where a punctum is considered co-localized even if a single pixel overlaps with the other channel, through >10%, >20% …, 90%, to =100%, where a punctum is considered co-localized only if its entire area is covered by a punctum of the other channel.

**Figure 7.**
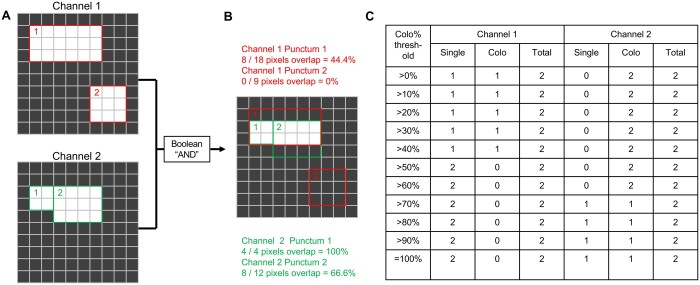
Co-localization analysis. Co-localization is based on percent overlap. (A and B) A Boolean “AND” operation is performed pixel-wise across the two channels of an image, resulting in an image where only pixels that were common to both channels remain white. The program then calculates the area in each punctum taken up by white pixels. A punctum is classified as “single” if the fraction of white pixels falls below the co-localization threshold and “colocalized” if the fraction is above the threshold. (C) The program tabulates the number of “single” puncta, “colocalized” puncta, sorted according to different thresholds from >0% to =100%.

To perform this analysis, NMJ Analyser opens the binary images representing the detected puncta generated by Otsu thresholding earlier for the two channels on a single slice of the image (Fig. 7A). A Boolean “and” operation is performed with both images as inputs. The result is an image that marks only the region where the two initial channels overlap (Fig. 7B). The area representing each punctum is overlaid onto the “and” image and the percent of each punctum covered by the other channel is calculated. NMJ Analyser that tabulates all the puncta into a table (Fig. 7C) that tallies up the number total number of puncta per channel as well as the number of single and co-localized puncta (Fig. 7C). Whether a punctum counts as a single or co-localized punctum depends on whether the fraction of its area covered by the other channel reaches one of the 11 predefined thresholds.

### 3.6 NMJ co-localization in WT and C9orf72 zebrafish ALS model

ALS is a fatal neurodegenerative disorder characterized by the selective loss of motor neurons. One of the primary pathological processes in ALS is the disruption of NMJ morphology, caused by dysfunction in both the pre- and postsynaptic components. The most common genetic mutation associated with ALS is the hexanucleotide repeat expansion GGGGCC (G4C2) found in the first intronic region of the *C9orf72* gene, present in 40% of familial ALS cases. We recently developed a zebrafish ALS model with knockdown of the *c9orf72* gene (hereafter referred as the C9-miR model) that replicates the characteristic features of ALS observed in patients (Butti *et al.* 2021).

**Figure 8.**
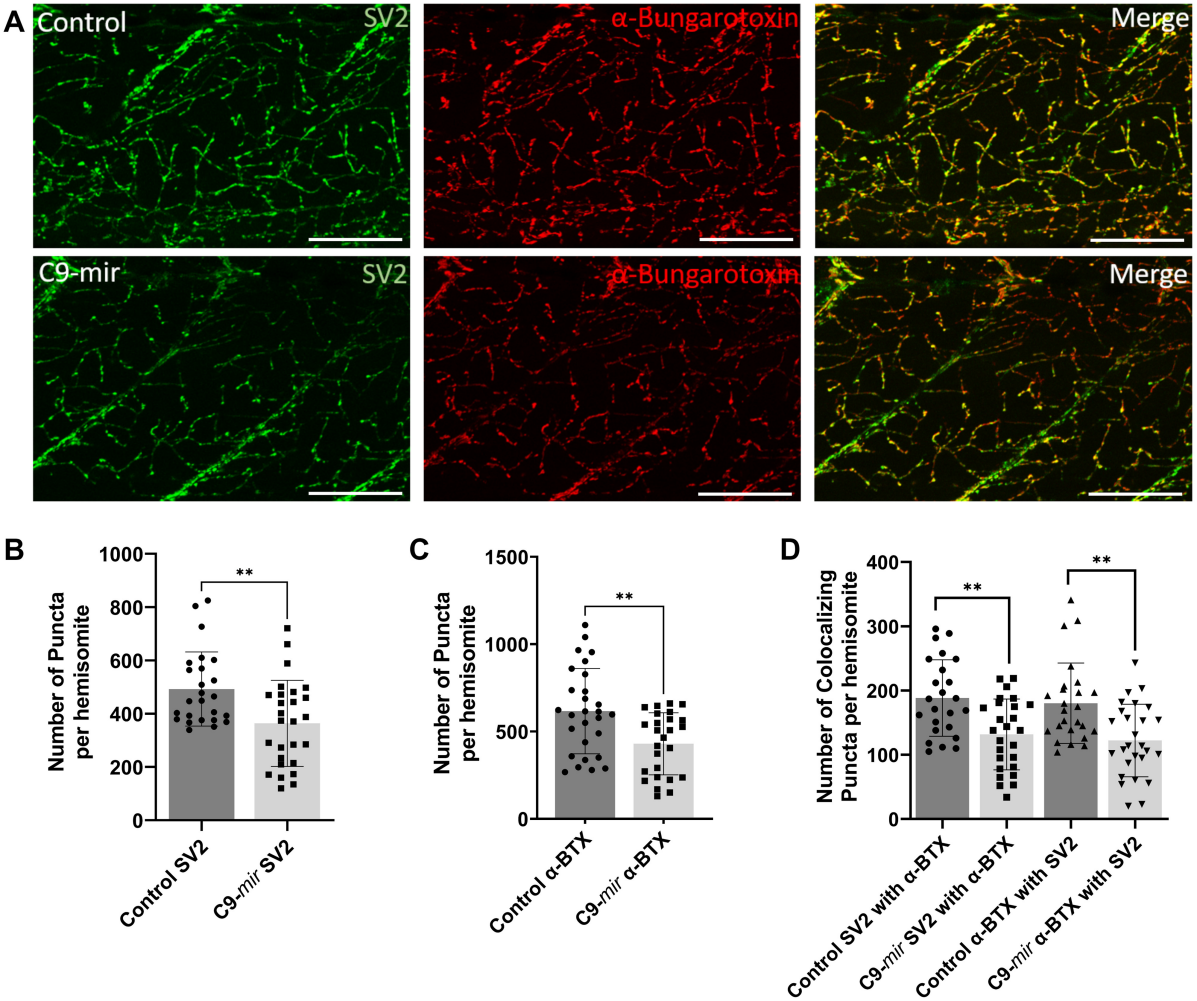
NMJ Morphology in Control versus C9-miR Zebrafish. (A) Co-immunostaining of zebrafish NMJs with presynaptic (SV2a; green) and postsynaptic (α-bungarotoxin; red) markers at 6 dpf. Scale bar = 100 µm. (B) Quantitative analysis demonstrated a significant decrease in the number of presynaptic (SV2a) puncta in C9-miR zebrafish (363.9 ± 161.7) compared to WT zebrafish (492.7 ± 139.2). (C) Quantitative analysis demonstrated a significant decrease in the number of postsynaptic (α-BTX) puncta in C9-miR zebrafish (430.0 ± 177.8) compared to WT zebrafish (616.9 ± 243.7). (D) Co-localization analysis revealed a significant decrease in the co-localization of presynaptic (SV2) puncta with postsynaptic (α-BTX) puncta in C9-miR zebrafish (131.7 ± 55.2) compared to WT zebrafish (188.4 ± 59.5). Similarly, co-localization analysis of postsynaptic (α-BTX) puncta with presynaptic (SV2) puncta revealed a marked reduction in co-localizing puncta in C9-miR zebrafish (122.1 ± 56.5) compared to WT zebrafish (180.3 ± 62.6). *n* = 24–28; **P < 0.001. Data are presented as mean ± SD. *n* represents the number of hemisomites.

The C9-miR zebrafish exhibited early motor impairments, characterized by decreased swimming distance and velocity. Furthermore, at 6 dpf, the C9-miR zebrafish displayed altered NMJ morphology (Fig. 8A). We employed our program to assess the NMJ morphology by automatically quantifying the presynaptic (SV2a), postsynaptic (a-BTX), and co-localizing punctae in WT versus C9-miR fish. Our analysis revealed a significant decrease in the number of presynaptic (SV2a) and postsynaptic (a-BTX) puncta in C9-miR zebrafish compared to WT zebrafish (Fig. 8B and C). Additionally, we observed a significant reduction in the co-localization of presynaptic (SV2a) puncta with postsynaptic (a-BTX) puncta, as well as a decrease in the co-localization of postsynaptic (a-BTX) puncta with presynaptic (SV2a) puncta in C9-miR zebrafish compared to WT zebrafish (Fig. 8D).

These results demonstrate the effectiveness of our program in accurately quantifying defects in NMJ morphology. The successful application of our program in assessing NMJ abnormalities in the context of ALS suggests its potential utility for quantifying NMJ defects in other NMDs, such as spinal muscular atrophy (SMA), Duchenne muscular dystrophy (DMD), Charcot-Marie-Tooth disease (CMT), myasthenia gravis (MG), and others. This highlights the broader applicability of our program in investigating NMJ defects in various neuromuscular disorders and advancing our understanding of their underlying pathogenic mechanisms.

## 4 Discussion

In this study, we present a specialized image analysis program for the analysis of zebrafish NMJ morphology. Zebrafish are widely utilized in the study of neurodevelopment and NMDs, and the analysis of NMJ morphology is one of the key indicators for motor function (Singh and Patten 2022). The generation of large amounts of data through genetic screening and high-throughput drug assays poses a significant challenge for the manual analysis of NMJ morphology, as it is a time-consuming task. Moreover, the limitations in the resolution of microscopes often result in images that introduce biases during manual analysis. To address these challenges, we have developed an automated image analysis program integrated into the open-source software Fiji (Schindelin *et al.* 2012). The program is designed to address the challenges of manual analysis and overcome the limitations of commercial solutions. By integrating the program into an open-source image analysis software Fiji, the method is made more accessible and applicable for researchers.

The program provides an automated workflow pipeline for confocal zebrafish NMJ files to create z-projections and allows users to define their ROI within the z-projection. The results of the analysis include the number of presynaptic and postsynaptic puncta, as well as the number of presynaptic and postsynaptic puncta co-localizing with each other. To ensure accurate analysis of NMJ morphology, we have incorporated an unbiased segmentation algorithm based on the analysis of three parameters, area, circularity, and aspect ratio, to detect and segment clustered puncta. Our program provides high-throughput, high-accuracy data analysis in a short time frame through an unbiased approach.

We applied our specialized image analysis program to assess the NMJ morphology in a zebrafish model of ALS induced by the knockdown of *c9orf72* gene. Our laboratory created a loss-of-function zebrafish model by targeted miRNA gene silencing approach, resulting in the specific and ubiquitous knockdown of the endogenous *c9orf72* gene expression (Butti *et al.* 2021). At 6 dpf, the C9-miR fish exhibited early motor impairments, including reduced swimming distance and velocity, and disruptions in NMJ morphology (Butti *et al.* 2021). Our program was used to quantify the NMJ defects in the C9-miR fish, demonstrating its utility in evaluating NMJ morphological defects in other NMDs, such as SMA, CMT, MG, and DMD etc.

In order to enhance the applicability and versatility of our program, we have incorporated an advanced mode. This mode empowers users to adjust various parameters according to their specific requirements. For instance, upon initiation of the program, users are presented with the option to either conduct image segmentation or skip this step. If they opt for segmentation, they can either utilize the default parameters of punctum analysis, which are optimized for Zebrafish NMJ as: (i) area >4, (ii) circularity <0.65, and (iii) aspect ratio >2.5. Alternatively, users can specify their own values for the parameters of area, circularity, and aspect ratio for each channel. This advanced mode will enable the analysis of not only Zebrafish NMJ morphology, but also other scientific images, including mouse NMJ morphology, different subcellular compartments, and surface receptors. This level of customization allows for an unbiased and time-efficient analysis of scientific images, increasing the utility and effectiveness of our program.

In conclusion, the development of the automated image analysis program for zebrafish NMJ morphology is a significant step forward in the study of NMDs and the use of zebrafish as a model organism. Additionally, the wide application of the program will provide a more efficient and accurate solution for researchers for image analysis and will open up new possibilities for the analysis of development and disease mechanisms.

## Supplementary Material

btad720_Supplementary_Data
